# CSF1R-related leukoencephalopathy: experimental models and potential for treatment

**DOI:** 10.1242/dmm.052957

**Published:** 2026-07-24

**Authors:** David A. Hume, Katharine M. Irvine

**Affiliations:** Mater Research Institute-University of Queensland, Translational Research Institute, Woolloongabba, Brisbane, QLD 4102, Australia

**Keywords:** CSF1R, CSF1R-related leukoencephalopathy, Adult-onset leukoencephalopathy with axonal spheroids and pigmented glia, Congenital microglial deficiency, CSF1R-related disorder

## Abstract

Dominant and recessive mutations in the human *CSF1R* gene are associated with microglial deficiency in the brain and severe neurodegenerative disease, known as CSF1R-related leukoencephalopathy (CRL). Dominant and recessive *Csf1r* mutations have been generated in mice, rats, zebrafish and chicken, providing models of the complete or partial microglial loss seen in patients. The impact of *Csf1r* mutations in inbred mice depends upon genetic background. For example, *Csf1r* mutants in the C57BL/6J strain are uniquely susceptible to perinatal mortality and hydrocephalus. Congenital microglial deficiency in a range of animal models does not influence postnatal brain development but is associated with age-dependent neuropathology that resembles CRL, indicating that microglial deficiency contributes to disease. None of the available models fully recapitulates the severe functional motor and cognitive impairments seen in patients, raising questions about species differences and the relative contributions of genetic and environmental modifiers. However, they have provided platforms to test ways to repopulate the brain with functional microglia. Here, we briefly review the genetic basis for CRL and evidence of variable penetrance. We also assess experimental models that can enable the development of therapeutic strategies.

## Teaser

**Summary:** Mutations in the *CSF1R* gene in humans are associated with severe neuropathology. Here, we assess the impact of CSF1R mutations in animals and their relevance to understanding human CSF1R-related disease.

## Introduction

The rare and fatal adult-onset leukoencephalopathy with axonal spheroids and pigmented glia (ALSP) is an autosomal dominant neurodegenerative disease associated with white matter destruction, rapid loss of motor function and cognitive decline (Dulski et al., 2024a; Konno et al., 2018; Papapetropoulos et al., 2024). A key discovery in 2011 linked ALSP to coding mutations in the *CSF1R* gene (Rademakers et al., 2011). The mechanisms linking CSF1R mutations to neuropathology have been reviewed by others (Chitu et al., 2022). In this Review, we provide an overview of the genetic basis of ALSP, also known as CSF1R-related leukoencephalopathy (CRL), highlight recent progress in the development of informative experimental models of CRL and discuss areas of disagreement that may influence approaches to therapy.

## Genetic basis for CRL

**Fig. 1.**
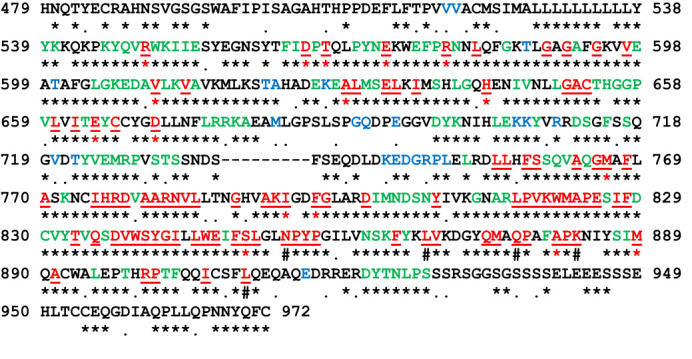
**Protein-coding variants in the CSF1R intracellular domain in patients and human populations.** The human reference CSF1R protein sequence is shown (ENSG00000182578). It was aligned with chicken CSF1R (ENSGALG00015021370), and asterisks indicate conserved amino acids. Coding variants present in aggregated population exome and genome sequences were identified in gnomAD v4.1. Amino acids affected by disease-associated mutations were identified from the Human Gene Mutation Database (HGMD). Red underline, amino acids affected by non-synonymous substitutions in CRL. Green highlight, conserved amino acids with rare (<10^−5^) non-synonymous substitutions in gnomAD, Blue highlight, amino acids with uncommon (>5×10^−4^) non-synonymous substitutions in gnomAD. Hash tags indicate disease-associated variants in non-conserved amino acids. Red asterisks indicate disease-associated variants also present in gnomAD.

*CSF1R* encodes a protein tyrosine kinase receptor that has two separate ligands: macrophage colony stimulating factor 1 (CSF1) and interleukin 34 (IL34) (Hume et al., 2020; Nandi et al., 2012). There have been reports that *Csf1r* can be expressed by neurons (Luo et al., 2013; Nandi et al., 2012), but analysis of a knock-in reporter transgene (Grabert et al., 2020), *in situ* mRNA localisation (Kana et al., 2019) and single-nucleus RNA-sequencing data (Kiani Shabestari et al., 2022) indicate that the gene is expressed exclusively in cells of the monocyte/macrophage lineage, including microglia and resident macrophages in the brain. According to the Human Gene Mutation Database (HGMD; June 2026), more than 200 different CSF1R coding mutations have been described in patients, the large majority giving rise to amino acid substitutions of evolutionarily conserved amino acids in the intracellular tyrosine kinase domain that is essential for transmitting the growth factor signal. Fig. 1 compares the protein sequence of this domain in humans to chicken CSF1R, which, despite the evolutionary distance, transmits a proliferative signal when expressed in a factor-dependent mouse cell line (Garceau et al., 2010). The alignment highlights the conserved amino acids in the CSF1R intracellular domain that have been the target of disease-associated coding variants. Interestingly, analysis of genomic DNA and exome sequences in gnomAD and UK Biobank (Wade et al., 2024) identifies many additional very-rare (<10^−5^) variants affecting conserved amino acids (see Fig. 1). They include tyrosine residues Y561, Y697, Y708, Y723 and Y809, which are phosphorylated when the receptor is activated and provide docking sites for downstream signalling pathways (Mouchemore and Pixley, 2012). Few known disease-causing variants are detected in gnomAD. There are two conclusions from these observations: (1) most disease-associated mutations are new mutations and private to individual families; and (2) the prevalence of carriers of potential disease-causing variants in CSF1R in the population – around 1:3500 based upon our analysis of the population data in gnomAD and the UK Biobank (Wade et al., 2024) – is probably around tenfold greater than the estimated disease prevalence (Papapetropoulos et al., 2021). The actual disease prevalence may be underestimated as there are documented cases of misdiagnosis of individuals with pathogenic CSF1R variants as other forms of dementia (Papapetropoulos et al., 2024). For example, three candidate pathogenic *CSF1R* variants were identified in a cohort of 465 patients with Alzheimer’s disease (Sassi et al., 2018), and novel variants have also been associated with a diagnosis of frontotemporal dementia (Pancaldi et al., 2026 and references therein).

Given that CRL disease onset typically occurs in postreproductive life, it is surprising that disease-associated variants are not enriched in particular ethnicities. Indeed, even the most common disease-associated variant, I794T, reported in ∼70 individuals, the majority from East Asia (Li et al., 2026), was shown to be present on two distinct genetic backgrounds (haplotypes) and has therefore arisen independently (Dulski et al., 2024a). By contrast, several substitutions that affect non-conserved CSF1R amino acids are relatively prevalent in European populations (e.g. E920D, G747R, G413S, H362R; gnomAD), consistent with a founder effect. Because CSF1/CSF1R signalling regulates both male and female reproduction (Cohen et al., 2002), it is possible that functional variants in the parents reduce overall fertility and, hence, the likelihood of transmission to subsequent generations. Alternatively, negative selection of CSF1R variants could arise through their potential impact on macrophage function in innate immunity and susceptibility to infectious disease. The possibility that coding variants in CSF1R confer increased susceptibility to infectious or inflammatory disease has not yet been sufficiently investigated, and the effect of dominant *CSF1R* mutation on peripheral neuronal function also warrants further consideration. For example, recent evidence links intestinal muscularis macrophages, which interact with and regulate enteric neurons in a CSF1-dependent manner (Muller et al., 2014), to the development of Parkinson’s disease. Muscularis macrophages can accumulate misfolded α-synuclein, the causative agent of Parkinson's disease, and modulate the expansion of T cells that traffic to the brain as Parkinson's disease-associated pathology progresses (De Schepper et al., 2026).

As with many other adult-onset diseases, CRL penetrance is likely to be influenced by a combination of genetic background (epistasis) and environmental effects. Genetic modifiers may include ethnicity, as recorded cases in China have presented, on average, at a younger age, and sex, with females presenting 5 years earlier than males (Dulski et al., 2024a; Li et al., 2026). Genetic variants that influence susceptibility to other more common forms of dementia are obvious candidate disease modifiers. Considerable advances have been made in the past 20 years towards understanding the genetic basis of susceptibility towards Alzheimer’s disease. At least 80 loci have been identified in genome-wide association studies, many containing genes expressed specifically in microglia (Heneka et al., 2025). *CSF1R* mutations could potentially interact with a susceptible genetic background to produce an early-onset neurodegenerative disease. Indeed, a recent study demonstrated that the *APOE4* variant linked to Alzheimer’s disease susceptibility was associated with accelerated progression in CRL (Chmiela et al., 2026a). Variation in expression or function of the two ligands for CSF1R likely also contributes to the risk of developing neuropathology. Loss-of-function mutation in rat IL34 was not dosage compensated (Huang et al., 2025), so haploinsufficiency (50% reduced expression) in heterozygotes affected microglial density. A relatively common stop gain mutation in *IL34* has been associated with increased risk of developing dementia (de Rojas et al., 2021; Hernandez-Rasco et al., 2026 preprint). The gnomAD database reveals multiple additional coding mutations affecting conserved amino acids that might compromise IL34 function but remain to be tested. Similarly, a common single-nucleotide polymorphism linked to the *CSF1* locus is associated with increased risk of developing Paget’s disease of bone (Albagha et al., 2010).

Few of the variants shown in Fig. 1, even those that are apparently disease associated, have been shown experimentally to impact CSF1R function. CSF1/IL34 binding to the receptor leads to dimerisation and autophosphorylation, which can be tested in cell-based assays *in vitro*. Wild-type (WT) and mutant receptors can be transfected into cell lines that do not express CSF1R. Receptors with mutations in the intracellular domain are expressed on the cell surface and bind CSF1; however, they lack kinase/autophosphorylation activity (Konno et al., 2018; Uden et al., 1999) and cannot support the growth of a factor-dependent cell line (Pridans et al., 2013). These assays could potentially be used in high-throughput scanning mutagenesis to identify all of the amino acids that are essential for CSF1R signalling. In addition, there are a small number of disease-associated variants described in the extracellular domain (Konno et al., 2018), but it is not clear whether these mutations alter cell surface expression or ligand binding.

Subsequent to the discovery of disease-associated dominant *CSF1R* mutations, several research groups have described even rarer individuals with homozygous recessive *CSF1R* mutations associated with earlier and more severe neuropathology as well as impacts on musculoskeletal development – a syndrome referred to as brain abnormalities, neurodegeneration and dysosteosclerosis (BANDDOS) (Guo et al., 2019; Guo and Ikegawa, 2021; Oosterhof et al., 2019). Given the common underlying genetic basis and continuum of pathological consequences largely impacting white matter, ALSP and BANDDOS can reasonably be considered as heterozygous/dominant and homozygous/recessive, or late- and early-onset forms of CRL or CSF1R-related disorder (Dulski et al., 2024b).

There is also an obvious analogy between CRL and the spectrum of pathologies associated with mutations affecting the *TREM2* gene. *TREM2* is more highly expressed by microglia than by other tissue macrophages (Summers et al., 2020), is inducible by CSF1 and regulates CSF1R signalling (Keshvari et al., 2024). Homozygous recessive mutations are associated with Nasu–Hakola syndrome (Zhou et al., 2023), which shares many features with BANDDOS. Furthermore, a heterozygous coding mutation in *TREM2* predisposes to early-onset dementia (Wang et al., 2022).

**Fig. 2.**
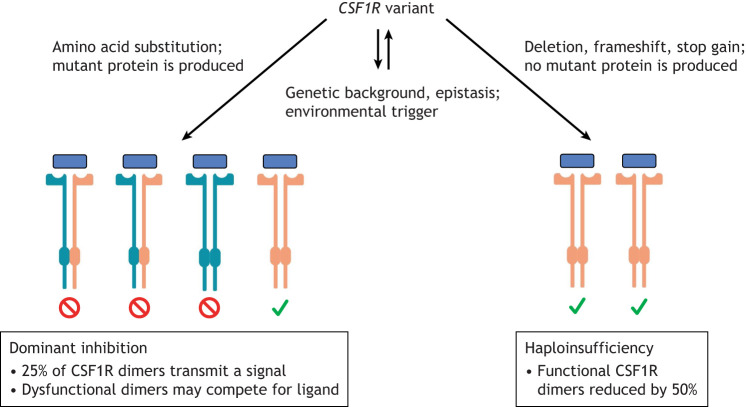
**Impacts of dominant and recessive *CSF1R* mutations.** When a heterozygous *CSF1R* variant causes an amino acid substitution and mutant protein is produced (blue), this leads to dominant inhibition. If the mutant protein dimerises with wild-type protein (orange), only 25% of dimers are functional, and they may be competing with dysfunctional dimers for the ligand. When a heterozygous *CSF1R* variant is a deletion, frameshift or stop gain variant and no mutant protein is produced, this leads to haploinsufficiency, with 50% functional CSF1R dimers.

Although the majority of dominant forms of CRL involve amino acid substitutions in the kinase domain, there are also many cases recorded in HGMD in which the mutant allele contains deletions, frameshifts, splicing defects or premature stop codons predicted to produce complete loss of CSF1R expression or function. This observation has led to the proposal that haploinsufficiency (i.e. 50% loss of activity) of CSF1R is sufficient to generate disease (Konno et al., 2018). It is certainly the case in animal models that heterozygous null *CSF1R* alleles are not dosage compensated, hence leading to 50% loss of protein expression (Patkar et al., 2021; Pridans et al., 2018; Rojo et al., 2019). An argument against haploinsufficiency as a strict causal mechanism is that carriers of recessive mutations do not appear to develop disease even in old age (Chitu et al., 2022). Monies et al. (2017) described siblings with severe brain malformation and generalised osteopetrosis associated with a definitive homozygous loss-of-function mutation in *CSF1R* (Y540*), which would remove the entire intracellular domain. No adverse phenotypes were observed in the parents or heterozygous siblings. Notably, in the initial description of patients with heterozygous null alleles and predicted haploinsufficiency, CSF1R protein in the brain was almost undetectable compared to in controls (Konno et al., 2018), suggesting >50% loss of expression. The effect of a heterozygous mutation on total CSF1R likely depends upon expression of the WT allele, which may be variable. Indeed, the expression of *CSF1R* mRNA in monocyte-derived human macrophages was reported to vary more than tenfold between individuals in a population cohort unrelated to CRL (O'Brien et al., 2024). The high prevalence of coding mutations in the tyrosine kinase domain favours a dominant inhibitory model in which the mutant proteins form dysfunctional dimers with the products of the WT allele. However, the dominant inhibitory and haploinsufficiency mechanisms need not be incompatible; the commonality is likely to be a sufficient reduction in CSF1R activity to increase the likelihood that an environmental stimulus leads to a neuropathological phenotype. The contrasting genetic models are summarised in Fig. 2.

Finally, as discussed below, the *CSF1R* gene contains an intronic enhancer that is more highly conserved across species than any of the exons and is required for expression in microglia (Rojo et al., 2019). The possibility that functional *CSF1R* enhancer variants exist and contribute to CRL is currently not considered in diagnostic exome sequencing.

Taken together, current evidence indicates that *CSF1R* coding variants cannot be considered as strictly causal for the development of neuropathology and are better described as conferring susceptibility. This conclusion is important for the development and interpretation of disease models.

## CSF1R mutations and the functions of microglia

**Fig. 3.**
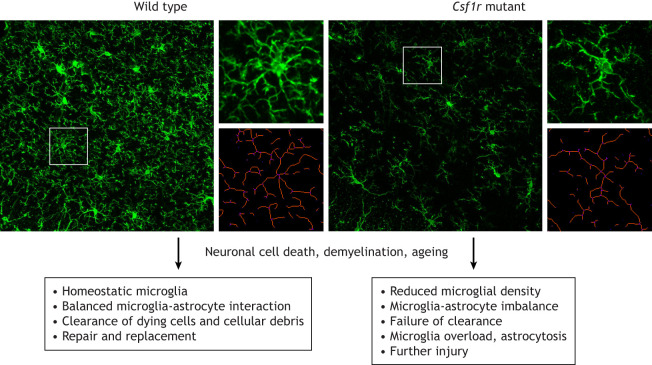
**The impact of CSF1R-related leukoencephalopathy-associated mutation on microglia in mice.** The images show the detection of the microglial marker IBA1 (green) in the cortex of adult wild-type and *Csf1r*^E631K/+^ mutant mice, highlighting the reduced density in the mutant. Insets show magnifications of individual microglia used and correspoding outline traces (red) to quantify the degree of ramification as described by Stables et al. (2022). Text below the images summarises the proposed mechanism linking CSF1R insufficiency to neuropathology.

Microglia, the major resident macrophages of the brain, participate in the establishment of neuronal connectivity, maturation of brain function and homeostasis in the adult brain (Depp et al., 2025; Hume, 2025; Paolicelli et al., 2022; Schafer et al., 2025). They are distributed regularly throughout every brain region, and each cell possesses an extended network of ramified membrane processes (Fig. 3). In the healthy adult brain, microglia have a distinctive gene expression profile compared to those of other tissue-resident macrophages (Summers et al., 2020). The so-called homeostatic microglial signature includes 200-300 transcripts that are expressed almost exclusively by microglia compared to all other brain cell types and are selectively depleted in microglia-deficient mice and rats (Elmore et al., 2014; Hume, 2025; Munro et al., 2024; Patkar et al., 2021). In many experimental models of neuropathology, the homeostatic microglial signature is repressed alongside increased expression of so-called damage-associated microglial transcripts (Depp et al., 2025; Paolicelli et al., 2022). Coding variants that impact the function of homeostatic microglia-expressed genes are associated with many monogenic and polygenic diseases affecting the brain, likely involving the accumulation of dysfunctional microglia and chronic inflammation leading to neuronal loss (Heneka et al., 2025; McKinney et al., 2026).

There is an intrinsic difference between mutations in *CSF1R* and other microglia-expressed transcripts, such as *TREM2*. CSF1R is absolutely required for the proliferation, differentiation and survival of microglia. Homozygous loss-of-function *CSF1R* mutations in humans (Guo et al., 2019; Oosterhof et al., 2019), mice (Erblich et al., 2011; Nandi et al., 2012), rats (Patkar et al., 2021) and zebrafish (Oosterhof et al., 2018) lead to complete microglial deficiency throughout embryonic and postnatal life. Immunolocalisation assessment of microglia in the brains of patients with dominant *CSF1R* mutations indicates reduced numbers, redistribution of remaining microglia to foci of apparent injury and the relative loss of homeostatic markers (Berdowski et al., 2022; Kempthorne et al., 2020). The relative loss of microglia and altered transcriptional profile are supported by transcriptomic analysis of mRNA isolated from postmortem patient brain tissue (Berdowski et al., 2022; Du et al., 2025). At autopsy, the absolute reduction in microglial cells reported in the brains of patients with dominant mutations is ∼50% (Berdowski et al., 2022; Du et al., 2025), but the extent of the reduction prior to disease onset is not known. The reduced microglial density in CRL brain might be linked to increased turnover, with higher rates of cell death only partly balanced by increased proliferation, a prediction confirmed in single-cell RNA-sequencing data in which proliferative microglia were enriched (Du et al., 2025). The relative loss of microglia is associated with increased abundance and activation of astrocytes, the most abundant glial population in the brain, and dysregulation of oligodendrocyte function (Berdowski et al., 2022; Du et al., 2025). Both CSF1R ligands, CSF1 and IL34, are internalised by receptor-mediated endocytosis (Stanley and Chitu, 2014), so the reduced steady-state density of microglia in CRL must cause an imbalance between ligand production and consumption. Neither *Csf1* nor *Il34* expression is altered in microglia-deficient mouse or rat brain at the mRNA level (O'Keeffe et al., 2025; Patkar et al., 2021). However, the major 4.5 kb *Csf1* transcript expressed in mouse brain (Thery et al., 1990) encodes a protein isoform expressed on the cell surface, which is released by ectodomain cleavage (Sehgal et al., 2021). It remains possible that reduced local CSF1 availability regulated at a post-transcriptional level contributes to the loss of microglia in CRL.

Many different experimental models of partial or complete microglial deficiency associated with CSF1R dysfunction have been generated since the original identification of *CSF1R* mutations in humans. These are summarised in Table 1 and discussed in detail below.

**Table 1.** Experimental models of CSF1R-related microglial deficiency

Model	References	Comments
Human iPSC	Chadarevian et al., 2024; Wong et al., 2025	Models impact of disease-associated mutations in cultured-derived microglia-like cells *in vitro*. iPSC-derived microglia can be transferred to humanised mice.
Mouse *Csf1rko*	Dai et al., 2002; Jacquelin et al., 2026; Li et al., 2006	Global macrophage and microglia deficiency. Severe postnatal impact depends on genetic background. Can be rescued by WT marrow transfer.
Rat *Csf1rko*	Patkar et al., 2021; Pridans et al., 2018	Global macrophage and microglia deficiency. Severe postnatal developmental impact. Ventricular enlargement. Can be rescued by WT marrow transfer.
Chicken *Csf1rko*	Wu et al., 2026	Global macrophage and microglia deficiency. Severe postnatal developmental impact. B cell deficient.
Mouse *Fireko*	Munro et al., 2024; Rojo et al., 2019	*Csf1r* enhancer deletion. Microglia deficient. Develops age-dependent neuropathology. Rescued by WT microglial transfer.
Heterozygous mouse *Csf1rko*	Arreola et al., 2021; Chitu et al., 2020; Chitu et al., 2015	Develops age-dependent microgliosis and neuropathology.
Heterozygous mouse *Csf1r* kinase domain mutation	Li et al., 2026; Stables et al., 2022; Stables et al., 2024; Wu et al., 2025	Disease-associated mutations. Reduced microglial density. Age-dependent pathology differs between models and laboratories.
*Il34ko* in mouse and rat	Huang et al., 2025; Wang et al., 2012	Reduced microglial density mainly in grey matter. No spontaneous pathology.
*Csf1r* mutations in zebrafish	Berdowski et al., 2022; Oosterhof et al., 2017	Complete or partial microglial deficiency with *Csf1rko* or dominant coding mutations.

iPSC, induced pluripotent stem cells; WT, wild type.

## Human induced pluripotent stem cell models

Induced pluripotent stem cells (iPSC) generated via expression of pluripotency-associated transcription factors in somatic cells have been widely applied to generate models of human genetic diseases. Several groups have published protocols for the generation of microglia-like cells from human iPSC (Banerjee et al., 2020; Chadarevian et al., 2026; Drager et al., 2022), and these cells are being tested as potential microglial replacement therapies (see ‘Treatment of CRL’ section). The availability of CRISPR-Cas9 editing enabled the introduction of defined disease-associated mutations or correction of mutations to create isogenic pairs of iPSC-derived microglia (Chadarevian et al., 2024; Wong et al., 2025). iPSC derived from two patients – one with the common I794T and one with a truncation mutation (R676Ter) – were recently used to generate an organoid model of CRL (Wong et al., 2025). A series of experiments suggested that macrophages generated following differentiation of the mutant cells exhibited an activated phenotype, compared to that of their isogenic controls, that in turn compromised neuronal cell function in co-cultures. A similar study reported the phenotype of iPSC derived from a patient with T567M mutation (Chi et al., 2026). This was of interest because the amino acid lies outside the kinase domain in the juxtamembrane domain. T567 is conserved in chicken CSF1R (Fig. 1), and other conserved amino acids in this region, including Y571, have been shown to be essential for kinase activity of mouse CSF1R (Myles et al., 1994). The T567M variant has been detected in multiple ethnicities, and several other disease-associated variants in the conserved juxtamembrane domain (R549H/C, E573K and D565N) are also relatively prevalent in human populations (minor allele frequency>10^−5^, gnomAD). These authors reported that the T567M mutation led to inflammatory activation of microglia derived from iPSC and consequent dysregulation of neurodevelopment in a cerebral organoid model (Chi et al., 2026). The use of iPSC as models is clearly a growth area, but the precise nature of pro-inflammatory stimulus in the culture systems and the mechanistic link to CSF1R signalling remains to be determined.

## Mouse models of complete microglial deficiency

The essential function of CSF1R in macrophage development and homeostasis was known well before the discovery of functional human *CSF1R* mutations in 2011. The mouse *Csf1r* gene was knocked out by homologous recombination in embryonic stem cells in 2002. The impact of the knockout (*Csf1rko*) on bone development in mice closely resembled the osteopetrotic phenotype associated with a natural mutation in the *Csf1* gene on a complex mixed genetic background (Dai et al., 2002). Subsequently, the *Csf1r* mutant allele was backcrossed to the inbred C57BL/6 and FVB backgrounds. On the C57BL/6J background, the *Csf1rko* was perinatal lethal (Nandi et al., 2012); on FVB and C57BL/6N backgrounds, the homozygous mutation led to complete microglia deficiency, but few pups survived to weaning owing to hydrocephalus (Erblich et al., 2011; Nandi et al., 2012). Despite the severe phenotype in inbred *Csf1rko* mice, adoptive transfer of WT bone marrow cells by intraperitoneal injection at birth was sufficient to enable population of the brain with microglia-like cells and rescued ∼50% of pups, resulting in long-term survival (Bennett et al., 2018). We recently generated an alternative homozygous *Csf1rko* based upon a CRL-associated kinase-dead *Csf1r* E631K mutation. The analysis of the heterozygous mutant mice as a model for CRL is discussed below. Homozygous *Csf1r* E631K mutation led to complete loss of macrophages in the embryo (Stables et al., 2022) and perinatal lethality (Jacquelin et al., 2026) on the C57BL/6 background, confirming the loss of CSF1R signalling activity. However, outcross to a mixed C57BL/6J×BALB/c F2 background fully rescued postnatal survival in homozygotes (Jacquelin et al., 2026). Homozygous E631K mutant mice on the F2 background largely recapitulated the original *Csf1rko*; they were osteopetrotic, growth retarded and lacked many tissue macrophages including microglia, but there was no ventricular enlargement or gross neurodevelopmental abnormality (Jacquelin et al., 2026).

The transcriptional regulation of *Csf1r* has been studied in detail to understand lineage commitment in the mononuclear phagocyte lineage (Rojo et al., 2017). Regulatory elements from the *Csf1r* locus have been used to generate reporter transgenes in mouse (Hawley et al., 2018; Sasmono et al., 2003) and rat (Irvine et al., 2020) that enable imaging of the location and dynamic behaviour of tissue macrophages (Petit et al., 2024). These studies identified a highly conserved enhancer element, the Fms intronic regulatory element (FIRE), that was essential for reporter gene expression (Sasmono et al., 2003). Homozygous deletion of this element in the mouse genome (*Fireko*) produced a line that was entirely deficient in microglia and selected tissue macrophages outside the brain, but, unlike the *Csf1rko*, was not osteopetrotic and grew normally (Rojo et al., 2019). Analysis of postnatal brain development of *Fireko* mice indicated that many functions previously considered to be dependent upon microglia, including myelination and synaptic maturation, occurred normally in their absence (McNamara et al., 2023; Munro et al., 2024; O'Keeffe et al., 2025).

The *Fireko* mice were generated on a mixed C57BL/6×CBA genetic background to avoid the potential lethality of the C57BL/6J background. Two other groups have regenerated the *Fireko* on distinct mixed genetic backgrounds (Baligacs et al., 2024; Wu et al., 2025). Backcross of the mutant allele to C57BL/6J revealed that the precaution was well founded as no viable *Fireko* pups were generated (Liu et al., 2026). We crossed the *Fireko* to a *Csf1r-*EGFP reporter line to enable imaging and, somewhat fortuitously, generated mice that retained small segments of non-C57BL/6J genomic DNA (Taylor et al., 2025 preprint). This congenic line still gave rise to significant perinatal loss, and around one-third of surviving *Fireko* pups developed hydrocephalus. Another group (Du et al., 2026) recently generated two *Fireko* lines by retargeting in C57BL/6J mice. Postnatal survival and incidence of hydrocephalus were similar to those in the *Fireko/Csf1r-*EGFP mice. It remains unclear how this retargeting selected for increased survival. To confirm the role of genetic background in the mouse phenotype, our C57BL/6J *Fireko* line was crossed to two other genetic backgrounds, CBA and BALB/c. In the F2 generation in both lines, *Fireko* pups were genotyped at the expected 1:4 Mendelian frequency and none developed hydrocephalus (Taylor et al., 2025 preprint). The *Fireko* backcrossed to the CBA genetic background is viable and has been deposited at The Jackson Laboratory (Strain #032783). Interestingly, the strain description at The Jackson Laboratory reports that homozygous mice are frequently born with small or missing eyes, and a majority of those mice exhibit a conjunctivitis-like phenotype. They suggest a possible interaction between microglial deficiency and the *Pde6b^rd1^* retinal degeneration mutation found in CBA/J inbred mice.

We conclude that perinatal survival and hydrocephalus are separate complex traits regulated by the many variants that distinguish C57BL/6J from other inbred strains (Hume, 2023). Among other idiosyncrasies, C57BL6/J mice have a *Csf1r* R554S mutation. This amino acid lies adjacent to a functional tyrosine residue (Yu et al., 2012), and S554 is conserved in all other mouse strains and mammals (ensembl.org). C57BL/6J mice are uniquely susceptible to mutations that cause hydrocephalus, notably those affecting the function of ependymal cells lining the ventricles of the brain (Taylor et al., 2025 preprint). Ventricular enlargement is one of the major pathological features of CRL, so although the mouse genetics may seem somewhat esoteric, an understanding of the regulatory network linking *Csf1r* to cerebrospinal fluid homeostasis in mice could point towards epistatic mechanisms in human disease.

Although there is little apparent impact on postnatal brain development, analysis of aged *Fireko* mice supports the view that microglial deficiency is sufficient to generate neuropathology that resembles CRL. This CRL-like neuropathology includes demyelination and disruption of white matter integrity, axonal spheroids, cerebral atrophy and neuronal loss, vascular alterations, severe thalamic calcification, dysregulation of astrocyte and oligodendrocyte function, and early mortality (McNamara et al., 2023; Munro et al., 2024). The dysregulated oligodendrocytes in the *Fireko* mouse closely resembled transcriptomic alterations in the brains of patients with CRL (Du et al., 2025). These pathologies were prevented by intracranial adoptive transfer of WT microglial cells (Munro et al., 2024). The fundamental conclusions were replicated in a humanised *Fireko* mouse model in which adoptive transfer of human iPSC-derived microglia prevented and even partly reversed the development of pathology (Chadarevian et al., 2024). In this model, iPSC generated from a patient with CRL had reduced repopulation capacity, which was restored by gene correction of *CSF1R*. The protective functions of microglia were further examined by crossing the *Fireko* mice to the 5xFAD model of Alzheimer’s disease. This combination led to a shift from parenchymal amyloid plaques seen in the 5xFAD model to cerebral amyloid angiopathy, increasing brain calcification and haemorrhages, producing premature lethality (Kiani Shabestari et al., 2022). Similarly, in an infectious prion disease model, the *Fireko* mice exhibited accelerated pathology associated with premature astrocyte activation (Bradford et al., 2022). Paradoxically, studies using *Fireko* mice indicated that microglia may be required for initiation of amyloid plaque formation while also constraining subsequent pathology (Baligacs et al., 2024). These studies highlight the complex roles of microglia in neuroinflammation (Heneka et al., 2025). In view of the extensive pathology in *Fireko* mouse brains, analysis of cognitive and motor function revealed surprisingly little adverse effect distinguishing WT and *Fireko* mice (McNamara et al., 2023; Munro et al., 2024; Taylor et al., 2025 preprint). The remarkable resilience and plasticity of rodents with extensive underlying neuropathology is shared with Alzheimer’s disease models (Ameen-Ali et al., 2017) and is an intrinsic constraint on the utility and interpretation of behavioural assays.

## Rat models of complete microglial deficiency

The laboratory rat has many advantages over the mouse as a model in physiology and behaviour, and there are significant differences in macrophage biology between the two rodent species (Hume et al., 2021). Homozygous null mutation in the rat *Csf1r* locus led to the loss of most tissue macrophage populations, osteopetrosis and postnatal growth retardation (Keshvari et al., 2021; Pridans et al., 2018), resembling BANDDOS. The brains of *Csf1rko* rats were microglia deficient, and lateral ventricles were greatly enlarged (Patkar et al., 2021; Pridans et al., 2018). Transcriptomic profiling of multiple brain regions enabled the identification of a microglia-specific signature (Patkar et al., 2021). Otherwise, brain region-specific transcriptomic profiles were largely unaffected, although the enlarged ventricles may have contributed to a brain-wide increase in detection of stress-response genes. The *Csf1rko* rats also developed an emphysema-like lung pathology and breathing difficulties at ∼8-12 weeks that required euthanasia. However, intraperitoneal adoptive transfer of WT bone marrow cells (BMT) at weaning enabled complete phenotypic rescue, including repopulation of the brain with microglia-like cells, achieving a similar density to that in WT brains (Carter-Cusack et al., 2025; Keshvari et al., 2021; Sehgal et al., 2023). Transcriptomic analysis of the re-populated brains after 12 weeks indicated that, as in the mouse (Bennett et al., 2018), the donor-derived cells have a distinct transcriptomic profile compared to that of homeostatic microglia derived from the yolk sac and fetal liver during normal development (Carter-Cusack et al., 2025), but there was no other indication of pathology.

Heterozygous *Csf1r* mutant rats were also generated, and the expression of CSF1R in macrophages was reduced by 50%, without any evidence of brain pathology up to 1 year of age (Patkar et al., 2021). The FIRE enhancer has also been deleted in the rat genome, leading to complete microglial deficiency (Green et al., 2026 preprint). Unlike *Csf1rko* rats, *Fireko* rats did not develop ventricular enlargement, but the effect of genetic background on this phenotype has not yet been explored. Like the *Fireko* mice, the *Fireko* rats develop thalamic calcification, but no significant defects were detected in a range of behavioural tests (Green et al., 2026 preprint).

A unique feature of the rat BMT experiments was that donor cell repopulation made no contribution to the circulating blood monocytes. Based upon these findings, we injected WT bone marrow cells into the peritoneal cavity of *Fireko* mice at weaning. The outcome was the same as in the *Csf1rko* rat, as donor cells completely repopulated vacant macrophage territories, including the brain, while making no contribution to circulating blood monocytes (Taylor et al., 2025 preprint). These findings raised the possibly that a macrophage-depleted peritoneal niche can sustain/expand a migratory macrophage progenitor population. This novel avenue of repopulation might provide an alternative to myeloablative BMT as a treatment for CRL.

## Mouse models of partial microglial deficiency

Based upon the proposal of haploinsufficiency as the molecular basis of dominant CRL, mice with heterozygous loss-of-function *Csf1r* mutation on the C57BL/6J background were generated as a disease model. The initial description of the model (Chitu et al., 2015) revealed that aged *Csf1r*^+/−^ mice developed many of the histopathological features of human CRL. By contrast to microglial deficiency in CRL, the mutant mice developed microgliosis – increased microglial density – alongside increased expression of inflammatory cytokines, suggesting a microglia-mediated inflammatory process. A subsequent study of this model from the same laboratory (Chitu et al., 2020) indicated that microglial dysregulation was dependent upon an imbalance between CSF1R and CSF2R signalling – a suggestion related to increased *CSF2* mRNA detected in the brain of patients (Kempthorne et al., 2020). The most recent study using this model linked heterozygous *Csf1r* mutation to reduced expression of metallothioneins and oxidative stress (Chitu et al., 2026).

The haploinsufficiency mouse model has been reproduced by others using various approaches (Arreola et al., 2021; Li et al., 2023). Heterozygous *Csf1r* mutant mice generated via cre-mediated deletion of a conditional *Csf1r* allele exhibited synaptic loss and disruption of extracellular matrix structures (perineuronal nets) that could be reversed by CSF1R kinase inhibitors (Arreola et al., 2021). The same conditional deletion model was reported as evidence that the microgliosis and associated behavioural impacts were specifically due to microglial dysfunction (Biundo et al., 2021).

One concern with these haploinsufficiency mouse models is that *Csf1r* mutation is not the only genetic difference between WT and mutant. The original *Csf1rko* mutation (Dai et al., 2002) and later conditional mutations (Li et al., 2006) were made in 129Sv embryonic stem cells, and the targeting vector was not removed. Impacts on closely linked genes, notably the adjacent *Pdgfrb* gene, or linkage to non-C57BL/6 variants, are not excluded despite backcross to C57BL/6J. The *Cx3cr1*-cre line used to generate conditional deletion of *Csf1r* (Arreola et al., 2021; Biundo et al., 2021) is a knockout, so the mutant mice are also heterozygous for CX3CR1 deficiency. The precise mechanism linking 50% loss of CSF1R to increased CSF2, reduced metallothionein (*Mt1*, *Mt3*) gene expression and microglial activation is unclear, but there may be an environmental component, as progression was reported to be accelerated by high-fat diet (Chitu et al., 2026, 2022).

Heterozygous *Fireko* mutation is not dosage compensated (Rojo et al., 2019). The effects of heterozygous mutation may be influenced by environment or genetic background. The heterozygous mutation on the original mixed background had no impact on microglial function in the University of Edinburgh facility (Munro et al., 2024) but caused microgliosis in mice in the UC Irvine facility that had been backcrossed twice to C57BL/6J (Stables et al., 2022). By contrast, our recent analysis of the heterozygous mutation on the congenic C57BL/6J background in the University of Queensland facility indicated reduced CSF1 responsiveness *in vitro* and delayed postnatal expansion of tissue macrophages, including microglia (Liu et al., 2026). We recently deleted a second conserved element in the mouse *Csf1r* locus, which we called the CS1FR upstream regulatory element-A (CUREA) (Maxwell et al., 2026). In a multicopy transgene, removal of CUREA abolished expression of *Csf1r* in a subset of microglia. In homozygous mutant mice (*Csf1r*^ΔCURE/ΔCUREA^), there was a selective 40-50% reduction in microglia in the cortex but no evidence of pathology (Maxwell et al., 2026).

To model the dominant coding mutations in CRL, the E631K and I792T [equivalent to human disease alleles E633K and I794T (Rademakers et al., 2011)] were introduced into the mouse genome (Stables et al., 2022). Transfection studies in a factor-dependent cell line indicated that the mutant proteins bearing these and other CRL-related kinase domain variants were expressed on the cell surface and bound CSF1 but failed to signal (Pridans et al., 2013). In mice, both mutations as heterozygotes led to reduced microglial density. The E631K variant was studied further in detail.

Our model to explain dominant CRL is that mutant proteins can form inactive heterodimers with the products of the WT allele. In principle, 75% of CSF1R dimers would be inactive and, furthermore, might compete for binding of the ligands with functional dimers (see Fig. 2). Consistent with a dominant-inhibitory mechanism, heterozygous E631K mutation (*Csf1r*^E631K/+^) reduced CSF1 responsiveness *in vitro* and *in vivo* and reduced microglial density (Stables et al., 2022). The effect on microglial density was greatest at 3 weeks of age and gradually resolved. Remaining microglia were still regularly distributed throughout the brain but were less branched than in WT brains (Fig. 3). The morphology of microglia in static images reflects the dynamic monitoring of their immediate environment by constant extension of membrane processes (Tieu et al., 2025). Processes of filipodia extension and motility are acutely regulated by CSF1/CSF1R (Chitu et al., 2005; Owen et al., 2007), so the altered microglial morphology in the mutant mouse likely reflects the inhibition of CSF1R signalling, which might, in turn, constrain the homeostatic monitoring functions of microglia. The heterozygous E631K mutation also impacted the postnatal development of macrophage populations in the periphery, notably including osteoclasts and the antigen-sampling CD169^+^ macrophages of the spleen (Stables et al., 2022).

Despite the reduced microglial density and diminished CSF1 responsiveness, *Csf1r*^E631K/+^ mice did not replicate the severe pathology of human CRL or the effect of complete microglial deficiency in *Fireko* mice. Behavioural sensorimotor analysis at 10 months of age showed no significant differences between WT and *Csf1r*^E631K/+^ mice (Stables et al., 2022). In transcriptomic analysis of the hippocampus and striatum at 12 and 43 weeks, the only significant alteration in *Csf1r*^E631K/+^ mice was a proportional reduction in detection of the microglial signature, consistent with the reduction in their density. The reduced expression of *Mt1* and *Mt3* or increased *Csf2* reported in *Csf1r^+/−^* mice (Chitu et al., 2026) was not evident at either age. Immunolocalisation of homeostatic markers P2RY12 and TMEM119 confirmed the lack of effect of the mutation on microglial protein production. There was no evidence of astrocyte activation or perturbation of oligodendrocyte function and no effect on age-dependent regulation of neuronal gene expression (Stables et al., 2024). No significant pathology was detected by either magnetic resonance imaging or histological examination at 15 months of age. Indeed, the differences in overall IBA1^+^ (also known as AIF1^+^) microglia and macrophage cell density seen in younger animals were no longer evident at 15 months. Disaggregation of the aged brains yielded similar numbers of microglia in *Csf1r*^E631K/+^ and WT mice, with unchanged profiles of the microglial markers CD11b (also known as ITGAM), CD45 (also known as PTPRC) and P2RY12 (Stables et al., 2022).

The *Csf1r*^E631K/+^ mice were used to explore the idea that pathology arises from an interaction between the *Csf1r* mutation, genetic background and environment (Stables et al., 2024). An epistatic interaction was observed between *Csf1r*^E631K/+^ and *Cx3cr1*^+/EGFP^ (heterozygous EGFP knock-in creating a null allele), leading to microglial dysregulation and changes in neuronal gene expression. In simple terms, this models a situation in which there are fewer microglia and those remaining are dysfunctional. In two models of induced microgliosis, prion disease and experimental autoimmune encephalitis, microgliosis was reduced in *Csf1r*^E631K/+^ mice, but this was not sufficient to alter disease severity or progression (Stables et al., 2024). In the model of experimental autoimmune encephalitis, *Csf1r*^E631K/+^ mice showed the same pattern of activation of immune-related genes as in WT.

Another research group has since independently generated *Csf1r*^E631K/+^ and *Csf1r*^I792T/+^ mice, which inexplicably exhibited increased CSF1R phosphorylation in the brain (Wu et al., 2025). The shared phenotype with our prior model is the reduced density and altered morphology of microglia, but the age-dependent consequences apparently differ. WT mice and the two mutants (*Csf1r*^I792T/+^ and *Csf1r*^E631K/+^) were compared at different ages in assays of motor and cognitive function. There were significant, yet small, declines in performance in the mutant mice, but the comparison did not involve strict littermate controls. Mutant mice had occasional axonal spheroids and foci of calcification. Similar lesions occur spontaneously in aged C57BL/6J mice (Tarrant et al., 2020), and the trajectory of their detection appeared to be similar in WT and mutant mice at older ages (12-16 months) (Wu et al., 2025). Global changes in gene expression in microglia and oligodendrocytes detected by single-cell RNA sequencing (Wu et al., 2025) contrast the limited transcriptomic alterations observed in the brain in the original *Csf1r*^E631K/+^ analysed at similar age (Stables et al., 2024). More recently, a second study of the *Csf1r*^I792T/+^ model described a more penetrant neuropathology phenotype in younger animals and significant mortality at ∼1 year of age that was mitigated by microglial depletion followed by intracranial transfer of WT microglia (Li et al., 2026). The behavioural phenotypes reported in these models are rather mild but contrast with the absence of behavioural phenotypes in microglia-deficient *Fireko* mice despite the more extensive neuropathology. The difference is likely attributable to the increased resilience associated with the mixed genetic background of the *Fireko* mice, which also mitigates pathology in the 5xFAD Alzheimer’s disease model (Soni et al., 2024).

In our opinion, the apparent differences in phenotypes observed with the same dominant *Csf1r* mutation in different laboratories and between *Csf1r*^E631K/+^ and *Csf1r^+/−^* mice are likely to be attributable to genetics and environment, including the precise mouse genetic background and the environment including housing, feed, intestinal microbiome and undetected infectious agents. As an illustration of the influence of environment, spontaneous colitis in mice with mutations associated with human inflammatory bowel disease (*Nod2*/*Cybb*) was dependent on the commercial source of C57BL/6J mice (Caruso et al., 2019). Regardless of the nature of the difference, the analysis of the *Csf1r*^E631K/+^ mouse shows that the mutation alone is not strictly causal and does not lead to microglial dysfunction. Integrating the results obtained from *Csf1r*^E631K/+^ and *Fireko* mice, we suggest that the only direct effect of the heterozygous mutant is to inhibit CSF1R signalling. The consequent reduction in microglia density and motility leads to impaired response to injury (Fig. 3).

This model leaves open the question of how reduced CSF1R activity might alter the response to an environmental trigger. Cell surface CSF1R is subject to ectodomain cleavage by ADAM17 (Hernandez-Espinosa et al., 2025; Qing et al., 2016). Toll-like receptor (TLR) agonists promote this cleavage, inhibiting CSF1-induced proliferation while at the same time providing a pro-survival signal (Sester et al., 1999, 2006, 2005). It is possible that reduced CSF1R expression renders microglia more sensitive to this switch. Among possible TLR agonists in the brain, proteolytic cleavage of the abundant astrocyte-expressed protein SPARCL1 (also known as HEVIN) was recently reported as a mediator of astrocyte-microglia communication signalling via TLR4 (Ramirez et al., 2026). Importantly, the implication of ectodomain cleavage is that activated microglia may no longer be dependent on CSF1R signalling for survival and, hence, may be resistant to depletion using kinase inhibitors.

## Non-mammalian models of CSF1R biology

Zebrafish and chickens have been widely used as model organisms in developmental biology, in particular enabling access, visualisation and manipulation of the embryo *in ovo*, which is more difficult in mammalian embryos *in utero*. Both models have been applied to the study of CSF1R function.

The fundamental biology of microglial development, function and activation in response to injury is conserved in zebrafish (reviewed in Campos-Sanchez et al., 2025). The transparency of zebrafish embryos enables non-invasive live imaging of the emergence and behaviour of macrophages and microglia (Herbomel et al., 2001). Zebrafish have been used to model several aspects of CSF1R biology related to CRL. The *Csf1r* gene is duplicated in zebrafish, resulting in two orthologues, *csf1ra* and *csf1rb*. In fish with a mutation in *csf1ra*, macrophages generated in the yolk sac failed to invade the tissues of the developing embryo (Herbomel et al., 2001). Analysis of an allelic series of mutations in the duplicated *Csf1r* loci in the zebrafish revealed progressive reduction in microglial density and complete loss in double knockouts (*csf1rko*) (Oosterhof et al., 2018). However, these *csf1rko* fish were viable and fertile. Aside from the selective loss of microglia-expressed transcripts, there was also no evidence of pathology in aged fish. Following on from this, *csf1ra^−/−^*/*csf1rb*^+/−^ fish were analysed as a model of haploinsufficiency (Oosterhof et al., 2018). The loss of three Csf1r alleles led to redistribution of microglia, but the proliferative response to neuronal injury and the homeostatic gene expression signature was unaffected. By contrast, introduction of heterozygous disease-associated mutations in the kinase domain of the *csf1ra* gene on a *csf1rb^−/−^* background led to a 50% reduction in microglia, whereas a heterozygous frameshift loss-of-function mutation in *csf1ra* on the same *csf1rb^−/−^* background had no effect. These observations support a dominant inhibitory model for CRL-associated mutations.

The two ligands CSF1 and IL34 are also conserved and functional in macrophage homeostasis in birds (Garceau et al., 2010; Wu et al., 2020). Development of *CSF1R* transgenic chicks enabled visualisation of the earliest appearance of committed progenitors in the yolk sac (Balic et al., 2014) and the ability of bone marrow cells injected into the embryo to populate the entire mononuclear phagocyte system (Garceau et al., 2015). Advances in technologies for genetic modification in the chick via manipulation of primordial germ cells (Meunier et al., 2025) were recently applied to generate a *CSF1R* knockout, *CSF1RKO*. *CSF1RKO* chicks, like their mammalian equivalent, developed and hatched normally despite the absence of macrophages, including the microglia (Wu et al., 2026). Analysis of these birds revealed almost complete loss of B lymphocytes, indicating an essential function of CSF1R-dependent macrophages in the lymphoid organ, the bursa of Fabricius. Interestingly, *Csf1rko* mice and rats are also partly B cell deficient (Carter-Cusack et al., 2025; Dai et al., 2002; Jacquelin et al., 2026). The mechanism is not known, and, to our knowledge, B-cell function has not been assessed in patients with CRL. *CSF1RKO* chicks failed to thrive and required euthanasia ∼6-9 days post hatch (Wu et al., 2026). The possible impact of heterozygous *CSF1R* mutation has yet to be tested. It is likely that the macrophage deficiency in these chicks could be rescued by *in ovo* transfer of bone marrow progenitors (Garceau et al., 2015). The generation of CRL disease-associated coding mutations in chicken *CSF1R* is now feasible and would provide a novel platform in which to further dissect disease mechanisms and potential therapies*.*

## Models with loss of function mutations in CSF1R ligands

Both CSF1R ligands, CSF1 and IL34, are expressed in the brain, and mutations in their encoding genes also provide models of partial microglial deficiency. In mice, *Il34* is expressed predominantly by neurons and is induced in the postnatal period (Chitu et al., 2016; Kana et al., 2019). *Il34* knockouts in mice (Greter et al., 2012; Van Hove et al., 2025; Wang et al., 2012) and rats (Huang et al., 2025) have reduced density of microglia and brain-associated macrophages, predominantly in grey matter. Analysis of the *Il34* knockout rat (Huang et al., 2025) suggested that the small number of residual microglia in the cortex are sustained by a gradient of CSF1 diffusing from the corpus callosum. CSF1 production by neurons may contribute to prenatal and postnatal patterns of microglial expansion and distribution (Bridlance et al., 2026); but, in the adult, CSF1 is produced mainly by glia, oligodendrocytes and astrocytes, and is required for maintenance of white matter microglia (Chitu et al., 2016; Kana et al., 2019). Mice lacking both IL34 and CSF1 in the brain were generated by combining *Il34* knockout with conditional deletion of *Csf1* using nestin-cre, leading to almost complete loss of microglia in all brain regions (Kana et al., 2019). Any pathology associated with this novel model does not appear to have been further investigated. These authors focused primarily on the selective depletion of microglia in the cerebellum of CSF1-deficient mice and reported alterations in cerebellar development, homeostasis and motor function. These changes were not replicated in *Fireko* mice or *Csf1rko* rats, which lack microglia in the cerebellum (Patkar et al., 2021; Rojo et al., 2017; Taylor et al., 2025 preprint). To our knowledge, there has been no evidence of spontaneous neuropathology in either *Il34* or *Csf1* mutant animals. However, consistent with a role for microglia in neuroprotection, *Il34ko* mice displayed increased sensitivity to neurotropic virus infection (Wang et al., 2012) and ineffective response to amyloid plaque deposition in a model of dementia (Hernandez-Rasco et al., 2026 preprint).

## Treatment of CRL

If CRL is viewed as a primary microglial deficiency, the functional microglial dysregulation may be a consequence, rather than a cause, of pathology. In this circumstance, strategies to overcome the CSF1R signalling deficiency to restore microglial density would be considered. Agonist antibodies against TREM2, which lies downstream of CSF1R and regulates CSF1R signalling (Otero et al., 2009), have been consider as therapies in Alzheimer’s disease (Schlepckow et al., 2023). One TREM2 agonist antibody, iluzanebart, was developed to treat CRL (Meier et al., 2025), but a phase II trial was discontinued owing to lack of efficacy. Because the heterozygous mutation only partly inhibits CSF1R and does not prevent microgliosis in disease models (Stables et al., 2022, 2024), the other obvious option to manipulate CSF1R signalling is to increase the availability of CSF1 or IL34 in the brain. This has not yet been tested directly.

Another therapeutic option is to more directly replace the depleted microglia. The major impediments to replacing or replenishing microglia are the blood–brain barrier and the relative ineffectiveness of conventional BMT to achieve chimerism of microglia (Loeb et al., 2023). An additional concern is that bone marrow-derived microglia-like cells have a distinct gene expression profile resembling the microglia associated with neurodegeneration in mouse models (Bastos et al., 2025; Bennett et al., 2018; Carter-Cusack et al., 2025; Du et al., 2026). There is some evidence, including the humanised *Fireko* mouse model, that brain-wide peripheral myeloid engraftment leads to neuropathology (Blurton-Jones et al., 2025 preprint; Hohsfield et al., 2020; Wang et al., 2025 preprint), although this was not evident in transplanted *Csf1rko* rats (Carter-Cusack et al., 2025). This concern may be obviated by the use of fetal liver (or in humans, cord blood) donors, which can apparently give rise to homeostatic microglia (Bastos et al., 2025). The difference in transcriptomic profile between bone marrow-derived microglia and homeostatic resident microglia is attributed primarily to ontogeny (Paolicelli et al., 2022). The current dogma based upon rodent models is that microglia are maintained by self-renewal, with minimal contribution of bone marrow-derived cells (Paolicelli et al., 2022). Emerging evidence of age-dependent accumulation of monocyte-derived microglia indicates that this may not be entirely true in mice (Kim et al., 2025). More importantly, the tracking of clonal expansion of somatically acquired mutations revealed extensive infiltration of the healthy human brain with bone marrow-derived cells (Belk et al., 2026 preprint). This crucial observation raises the question of whether the reported impact of CRL-associated mutations on peripheral monocyte differentiation (Hofer et al., 2015) could contribute to microglial deficiency.

Notwithstanding the potential concerns, multiple recent publications have tested various approaches for microglial replacement, using WT bone marrow cells or monocytes as donors, as therapeutic solutions for other genetic diseases that are unequivocally linked to microglial dysfunction (Frosch et al., 2025; Mader et al., 2025; O'Brien et al., 2025; Tsourmas et al., 2025). As noted above, complete donor chimerism can be achieved without conditioning if the microglial niche is vacant, as in *Csf1rko* and *Fireko* mice and rats. This is because the ability of donor cells to populate the brain is likely to be constrained by competition for growth factors with the remaining recipient microglia. Furthermore, *Csf1r* mRNA is expressed at fivefold to tenfold higher levels in microglia than in blood monocytes (Summers et al., 2020). A novel solution is to generate microglia from iPSC that have mutations conferring resistance to a CSF1R kinase inhibitor, enabling selective depletion of the microglia of the recipient (Chadarevian et al., 2026; Lombroso et al., 2026). This aims to expedite engraftment, but this treatment alone is relatively ineffective owing to the rapid repopulation by endogenous microglia (Elmore et al., 2014). Multiple cycles of CSF1R kinase inhibitor treatment may exhaust repopulation activity and create a vacant niche to enable microglial transplantation (Chen et al., 2025; Du et al., 2026). As an alternative experimental model, deletion of *Csf1r* with tamoxifen-inducible cre recombinase was used to create a niche enabling monocyte engraftment (Bastos et al., 2025). The orally available CSF1R kinase inhibitor PLX5622 has also been very widely employed as a tool to investigate the functions of microglia in development and pathology (Green et al., 2020). However, at least some of the impacts of PLX5622 may arise from off-target activity on brain endothelial cells (Profaci et al., 2024) and potent activity as an agonist of the hepatic constitutive androstane receptor (Cao et al., 2026), which could compromise clinical applications.

Effective repopulation of the brain in *Csf1r*^I792T/+^ mice by donor-derived cells was achieved following myeloablative conditioning with busulfan prior to BMT (Wu et al., 2025). Microglia-deficient mice may be particularly amenable to the myeloablative approach because busulfan itself depletes ∼50% of microglia and renders the remainder senescent (Sailor et al., 2022). BMT appeared to mitigate subsequent development of pathology, but the effectiveness to alleviate pathology that was already established in older animals was not tested, and the complication of allogeneic donors and graft-versus-host disease in the brain was not considered. Furthermore, busulfan may have other toxic effects in the brain, including the complete inhibition of adult neurogenesis (Sailor et al., 2022).

Like most of the literature on monocyte-macrophage homeostasis, the majority of studies of microglia replacement employ C57BL/6J mice, and, as discussed above, the prevailing dogma is that there is minimal contribution from bone marrow-derived progenitors to microglia populations during the life course. This viewpoint is based upon fate-mapping studies that likely underestimate the extent of monocyte infiltration (Hume, 2023). Even in C57BL/6J mice, the ability of monocyte-derived microglia to seed the brain progressively during healthy ageing (Kim et al., 2025) raises the question of whether the extent of such infiltration depends upon mouse genetic background and environment, whether it is compromised by disease-associated CSF1R mutation in humans or mouse models, and whether it might be promoted by treatment with CSF1, which can produce a selective monocytosis (Keshvari et al., 2024).

There have been several published case series of conventional BMT aimed at microglial replacement in patients with CRL, with evidence of clinically meaningful delay or, in some cases, arrest of disease progression (Chmiela and Wszolek, 2025; Chmiela et al., 2026b; Tipton et al., 2021; Yska et al., 2025). The extent of microglial replacement/replenishment that was achieved in these patients is not clear. ^18^F-fluorodeoxy glucose positron emission tomography imaging was proposed as a surrogate marker of relative microglial abundance on the assumption that glucose uptake is enriched in microglia (Wu et al., 2025). Marginal increases in signal were detected in patients following BMT. However, *Slc2a1*, encoding the major glucose transporter expressed in the brain, is not significantly reduced in any brain region in microglia-deficient mice (Elmore et al., 2014; O'Keeffe et al., 2025; Taylor et al., 2025 preprint) or rats (Patkar et al., 2021). Conditioning strategies to deplete microglia, such as CSF1R kinase inhibitors, may have the potential to improve engraftment, but there have been few studies of pharmacology, safety or efficacy in any species other than mouse. Given the partial vacancy of the microglial niche, and diminished CSF1R signalling, it is possible that patients with CRL would have a reduced requirement for microglial depletion and that they could be more sensitive to potential toxicity. PLX3397 (pexidartinib), which was used in the cyclic microglial depletion model (Chen et al., 2025), is approved for the treatment of giant cell tumours. Liver toxicity associated with extended use (Lewis et al., 2021) may not necessarily constrain the short-term use to achieve microglial depletion, but the effectiveness needs to be tested in large animals before progressing to application in humans. For this purpose, the domestic pig may provide a human-relevant model for analysis of microglial function (Shih et al., 2023).

## Conclusions

A review of the field in 2022 was entitled “Modeling CSF-1 receptor deficiency diseases – how close are we?” (Chitu et al., 2022). The answer in 2026 would still be equivocal. The available rodent models do reproduce partial or complete loss of microglia as seen in the dominant or recessive forms of CRL in humans and provide useful platforms to test novel approaches to restore microglial homeostasis, but they do not mimic the rapidly progressive, ultimately lethal, human disease and hence have limitations for assessment of therapeutic efficacy. In part, this may simply be because inbred mice are not humans, and there are major differences in macrophage transcription and effector function between the species (Schroder et al., 2012). For instance, IL34 is more widely expressed in humans and rats than in mice (Huang et al., 2025). The relative lack of expression of *Csf1* in macrophages themselves is unique to mice (Sehgal et al., 2021), and, an alternative growth factor, FLT3L (also known as FLT3LG), regulates monocytopoiesis in humans but not in mice (Momenilandi et al., 2024). Furthermore, the impact of mouse genetic background provides evidence that genetic epistasis can modify the effect of *Csf1r* mutations. C57BL/6J mice differ from other mouse strains in many aspects of macrophage function and homeostasis (Hume, 2023) and appear uniquely susceptible to CSF1R-associated pathology. Finally, it seems likely that there is an environmental trigger to which individuals with CSF1R deficiency are uniquely susceptible. Given the functions of macrophages in immunity, the obvious candidates are infectious agents. As suggested elsewhere, Epstein–Barr virus is a plausible candidate known to express a virulence protein that represses CSF1R (Hume, 2025). If this was shown to be the case, treatments aimed at mitigating such infections might have therapeutic effects.

For the future, there are many outstanding questions:

1. Is the effect of heterozygous *Csf1r* mutation on microglial function cell autonomous or does it modify the response to another stimulus?
2. Is there a functional difference between 50% reduction in protein expression (haploinsufficiency) and expression of a mutant kinase-dead protein in patients or experimental models?
3. What components of the genetic background influence the likelihood of developing neuropathology in humans and experimental animals?
4. Is there a specific environmental trigger of disease in humans?
5. Are there effects of CSF1R on monocyte–macrophage function in the periphery that might contribute to disease and might also provide biomarkers?
6. Can the pathology of CRL be reversed?

Perhaps the most crucial question influences approaches to therapy. What is the function of microglia in disease? If microglia are mediators of pathology, their depletion would be desirable. If they are protective, their deletion could be harmful. The answers may provide a rationale for development of better models and improved treatments that extend beyond CRL to more common neuropathologies involving microglial dysfunction.
